# Modulators for Terahertz Communication: The Current State of the Art

**DOI:** 10.34133/2019/6482975

**Published:** 2019-05-29

**Authors:** Z. T. Ma, Z. X. Geng, Z. Y. Fan, J. Liu, H. D. Chen

**Affiliations:** ^1^College of Science, Minzu University of China, Beijing 100081, China; ^2^School of Information Engineering, Minzu University of China, Beijing 100081, China; ^3^State Key Laboratory for Integrated Optoelectronics, Institute of Semiconductors, Chinese Academy of Sciences, Beijing 100083, China; ^4^State Key Laboratory of Superlattices and Microstructures, Institute of Semiconductors, Chinese Academy of Sciences, Beijing 100083, China

## Abstract

With the increase of communication frequency, terahertz (THz) communication technology has been an important research field; particularly the terahertz modulator is becoming one of the core devices in THz communication system. The modulation performance of a THz communication system depends on the characterization of THz modulator. THz modulators based on different principles and materials have been studied and developed. However, they are still on the way to practical application due to low modulation speed, narrow bandwidth, and insufficient modulation depth. Therefore, we review the research progress of THz modulator in recent years and evaluate devices critically and comprehensively. We focus on the working principles such as electric, optical, optoelectrical, thermal, magnetic, programmable metamaterials and nonlinear modulation methods for THz wave with semiconductors, metamaterials, and 2D materials (such as graphene, molybdenum disulfide, and tungsten disulfide). Furthermore, we propose a guiding rule to select appropriate materials and modulation methods for specific applications in THz communication.

## 1. Introduction

**Figure 1:**
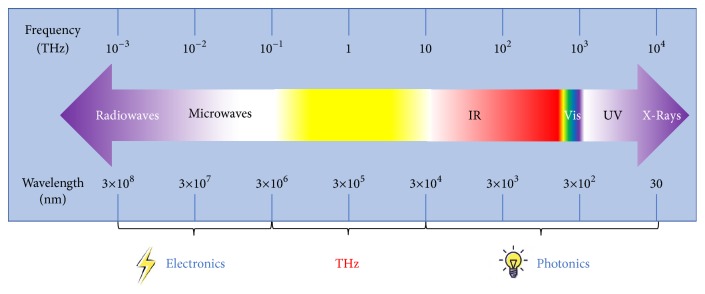
THz wave position in the electromagnetic spectrum.

The terahertz (THz) wave is firstly used to describe the spectral line frequency coverage of Michelson interferometer by Fleming in 1974 [1]. It is referred to as the “THz gap” in the electromagnetic spectrum until the mid-1980s [2, 3] due to the lack of effective methods for generating and detecting THz radiation. With development of femtosecond laser, the technology of THz wave generation and detection has been improved [4]. Generally, THz wave is referred to as the frequency range from 0.1THz to 10THz (corresponding to wavelengths between 30 *μ*m and 3 mm), which is the field of the transition from electronics to photonics [5, 6]. THz wave position in the electromagnetic spectrum is shown in Figure 1. THz wave occupies a crucial frequency range, because it usually carries vital physical information [7] which can be used in cutting-edge technologies [8, 9] such as wireless high-speed communications [10, 11], biomedical diagnostics [12–14], security imaging [15, 16], signal detection [17], and product quality control [18, 19]. However, the existing optical and microwave theories are unsuitable for THz wave perfectly [8].

In particular, THz wave is responsible for communications to achieve 10 Gbps wireless transmission speeds that are 100 to 1000 times faster than the current ultrawideband (UWB) technologies. Naturally, THz band as unoccupied spectrum resources is new choice for communication. THz communication has the merits which combined that of microwave communication and optical communication [20–22]. (1) The frequency of THz wave is 1~4 orders higher than microwave communication, which exhibits a larger communication capacity. (2) The narrow beam and good directivity of THz wave lead to strong anti-interference ability and high security. (3) THz wavelength is relatively short. Therefore, the size of antenna is small, which makes THz communication system relatively simple and compact. THz communication has maybe grown up as next generation communication technology [23–26]. To clarify the differences among THz wave, microwave, and visible-infrared light in communication, the comparison of different carrier communication characteristics is presented in Table 1.

As seen from Table 1, it illustrates that THz wave technology is equivalently suitable for communication occasions with various special requirements such as close-range secure communication and space-based communication [27, 28]. In THz communication system, modulators play a key role. Consequently, it has become a “hot” research field. More and more materials have been used to develop THz modulation devices currently, such as photonic crystals, metamaterial structures, phase change materials, high electron mobility transistors (HEMTs) structures, and graphene.

After reviewing and comparing recently developed THz modulators, we presented merits and drawbacks of different THz modulators. These THz modulators were classified by various approaches such as electronic, optical, photoelectric, thermal, magnetic modulation. The materials used in modulators focus on semiconductors, two-dimensional (2D) materials, and metamaterials. At last, the future development direction and application of THz modulator was proposed.

## 2. THz Modulation Technique

**Figure 2:**
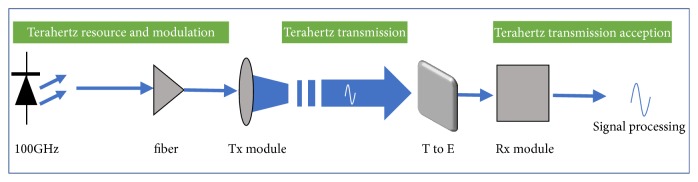
Diagram of THz communication.

The THz communication mainly depends on the THz modulation and demodulation technology, THz detection and reception technology, and THz generation technology. The rational use of modulators can effectively reduce the complexity, cost, and geometry of THz systems. Consequently, modulation technology is the focus of research in THz communication technology. Signal modulation refers to a process of using the modulation signal to control one or more parameters (amplitude, phase, etc.) of the carrier signal [29]. In recent years, various THz modulators based on different materials and structures [30–32] have been reported to achieve large modulation depth, fast modulation speed, and wide modulation bandwidth [33].  Figure 2 shows a diagram of the elementary THz communication [9].

### 2.1. Optically Tuned THz Modulator

The earliest THz modulator is based on hybrid multiquantum well structure, which is optically tuned to realize THz modulation [34]. Optical modulation often used a laser as the excitation light source. The laser irradiates the substrate material to generate carriers which can affect the conductivity of the material. The change in conductivity changes the transmittance and reflectivity of the THz wave transmitting in the material, which realizes the modulation of THz wave [35, 36].

#### 2.1.1. Modulator Based on Semiconductor Materials and Metamaterials

The semiconductor materials and metamaterials can artificially control the electromagnetic waves by designing the reasonable structure and size of unit cell [37]. In 2004, Tanaka et al. used 2D metal hole arrays (2D-MHAs) as a THz modulator [38]. Inspired by this work, many research groups began to use laser to modulate the THz wave. In THz wave region, the electric field response mechanism of conventional semiconductor materials can be explained by the Drude model [39] which treats free carriers in semiconductor materials as free electron gas. When laser irradiates on semiconductor materials, free carriers may be generated so long as the energy of the photon *hω* is greater than the bandgap of the semiconductor *E*_*g*_. The equivalent relative dielectric constant is given as

(1) $$\begin{array}{c} \varepsilon_{eff}\left(\omega \right)=1-\frac{\omega_{p}^{2}}{\omega^{2}+j\Gamma \omega } \end{array}$$

The plasma frequency is given as

(2) $$\begin{array}{c} \omega_{p}=\sqrt{\frac{Ne^{2}}{\varepsilon_{0}m_{eff}}} \end{array}$$

where Γ is related to the propagation loss; *ω*_*p*_ is the electron plasma frequency; *N* is the electron density; *e* is the electron charge; *m*_*eff*_ is the effective mass of the electron.

For semiconductor materials at normal temperature, microwave or THz wave transmitting in the material will be strongly attenuated, and it cannot even propagate. For the ultraviolet or higher band of *ω* ≥ *ω*_*p*_, materials are almost “transparent.” To obtain an electric field response in THz wave, it is necessary to lower the electron plasma frequency *ω*_*p*_ of materials. From the expression of plasma frequency, it is well known that the electron plasma frequency of materials can be varied by changing the effective mass and density of the electron. Therefore, using a laser with an energy greater than the forbidden bandwidth of materials can modulate the THz wave.

A THz modulator by exciting free electrons in a hybrid multiple-quantum-well structure was demonstrated by Libon et al. The electron density could reach 10^11^ cm^−2^ in a periodic quantum well structure. It realized a modulation depth of 40% in the range of 0.2-1 THz [34]. This was the first demonstration of optically tuned THz wave. In subsequent following research, semiconductor materials commonly used include high-resistance Si, high-resistance GaAs, and Si on sapphire. Meanwhile, combined with some structural design, such as photonic crystal, anisotropic medium, and surface plasma array, modulator could achieve better modulation effect. The modulator used in Li's work was a high-resistance Si wafer [40]. Unfortunately, the modulation speed was only 0.2 kHz, given that the lifetime of carriers in ultra-high-resistivity Si wafer was long. On the contrary, the lifetime of carriers in GaAs is short. Fekete et al. reported a method of embedding a GaAs defect layer in alternately stacked SiO_2_ and MgO periodic structures to constitute a one-dimensional photonic crystal. The efficient modulation of the THz beam can be achieved even at low photocarrier concentrations by exciting the front GaAs surface via ultrashort 810 nm laser pulses [41].

**Figure 3:**
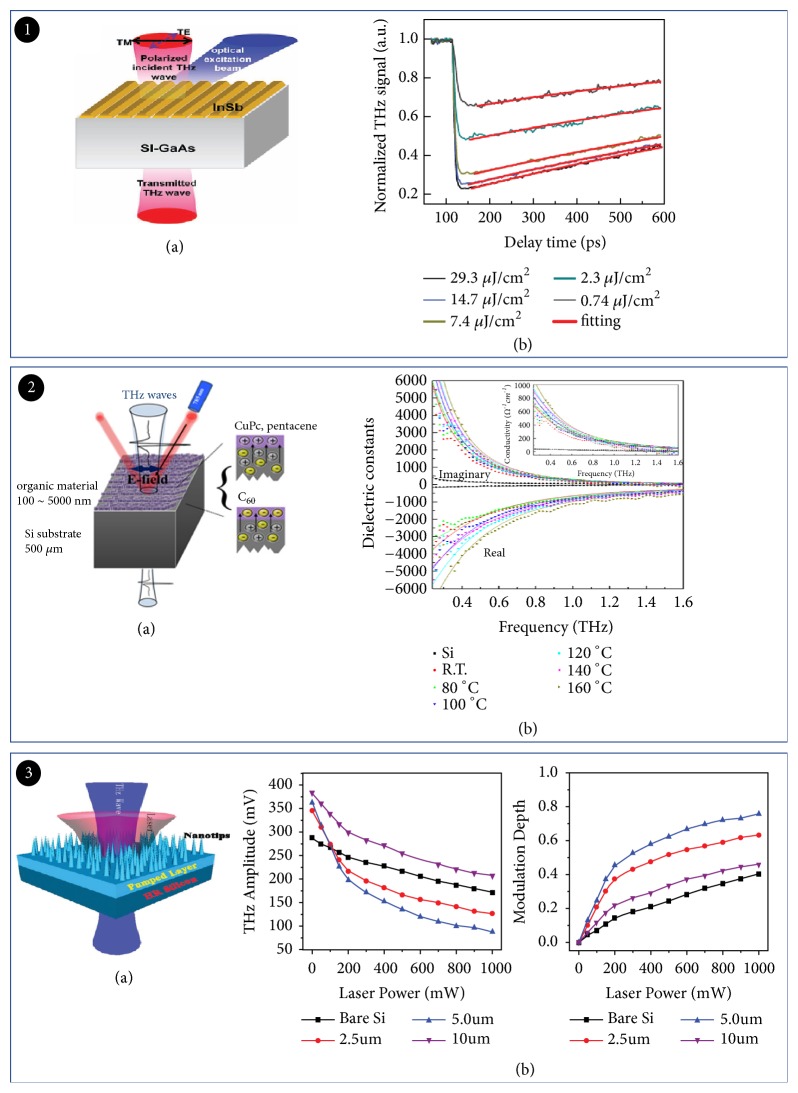
*Optically tuned THz modulator based on semiconductor materials and metamaterials*. Panel (1). (a) SEM images of the fabricated InSb grating. (b) Normalized THz signals as a function of delay time measured by OPTP under different pump laser fluences and the exponential curve fittings. Reproduced from [42]. Panel (2). (a) Schematic view of THz wave transmission measurement for bilayer samples. (b) Dielectric functions obtained from pentacene/Si thermally annealed at various temperatures. Reproduced from [43]. Panel (3). (a) Prototype and spatial configuration of the Si-nanotip-based spatial THz modulator. (b) THz transmission amplitude (left) and the modulation depth (right) of different modulators as a function of the laser pumping power. Reproduced from [44].

Theoretically, the modulation speed of the THz wave can reach to GHz. The difficulty lies in the carrier lifetime of the semiconductor substrate material. Usually, high laser energy can also solve this problem. The pump laser dynamically modifies the plasma frequency of the localized surface plasmon, rather than changing conductivity. In 2013, Deng et al. experimentally demonstrated the modulation speed of 1.2 GHz on InSb gratings fabricated on semi-insulating GaAs substrate (Figure 3(1)). This provided more creative possibilities for optical tuning in future THz devices [42]. Modulation efficiency depends on the energy level of the material, the crystal arrangement of the organic molecules, and the transported carriers at the interface. Actually, the active modulators can implement near-perfect modulation efficiency for THz communication applications [43]. Yoo et al. used a hybrid dual-layer system consisting of molecular organic semiconductors and Si to implement optically controlled active THz modulator (Figure 3(2)). The 98% modulation efficiency was achieved due to the rapid light-induced electron transfer from Si to C_60_ layer, which is almost fully modulated. At present, some studies have shown that the deposition of organic film on Si can also achieve full modulation [45, 46]. Since nanomaterials have unique physical properties, semiconductor nanostructures become a research hotspot [47]. The modulator used in Shi's work was a Si nanotip (SiNT) array (Figure 3(3)). The nanotip could be used as a THz wave antireflection layer to achieve low loss. This optically driven THz modulator with low loss and high modulation depth has the potential to be used in THz imaging [44].

The negative refractive index metamaterial is mainly realized by lattice of thin wires and open resonant rings. The unique electric field response of lattice of thin wires can achieve a negative dielectric constant; accordingly, the magnetic field response of the open resonant rings can achieve a negative magnetic permeability [48, 49]. Like the THz metamaterial modulator reported by Padilla, the modulator consists of a metal open resonant ring array structure on a GaAs substrate. When laser is illustrated on the GaAs substrate, photogenerated carriers affect opening capacitance with varying laser power. Thus, the transmission intensity of the THz wave would be modulated [50]. ‘‘Metasurfaces” are the two-dimensional version of metamaterials. Compared to three-dimensional bulk metamaterials, the thickness of the metasurfaces relative to the operating wavelength is negligible. They consume less physical space and have lower insertion loss. Longqing Cong et al. demonstrated an active hybrid metasurface integrated with patterned semiconductor inclusions for all-optical active control of terahertz waves [51]. It achieved ultrafast modulation of the polarization state. By properly incorporating silicon islands into the metamaterial unit cells, Xieyu Chen et al. presented a metasurface which can be optically controlled with a modulation depth reaching 68% and 62% for horizontal and vertical polarizations [52]. It opens up new avenues for the design of active metamaterials.

#### 2.1.2. Modulator Based on 2D Materials

2D materials have unusual electrical and optical properties. In recent years, they attracted increasing attention for applications in optoelectronics. Graphene, the best-known 2D material, has been widely used for THz modulators tuned by light since being discovered in 2004 [53]. The carbon atom arrangement of graphene determines its unique conical band structure. The conductivity of graphene is contributed by the in-band transition of electrons and the transition between bands. In the THz range, the in-band transition of electrons plays a decisive factor due to the photon energy being small. Therefore, we can approximate the thin layer conductivity of graphene by the Drude model as [54]

(3) $$\begin{array}{c} \sigma \left(\omega \right)=-\frac{jD}{\pi \left(\omega -i\Gamma \right)} \end{array}$$

*D* is the Drude weight, and it is given as

(4) $$\begin{array}{c} D=\frac{v_{F}e^{2}}{\hbar } \end{array}$$

(5) $$\begin{array}{c} v_{F}=\frac{E_{F}}{\hbar \sqrt{\left|n\right|}} \end{array}$$

where Γ contributed to the acoustic phonon scattering; *v*_*F*_ is the Fermi velocity; *n* is the carrier concentration.

**Figure 4:**
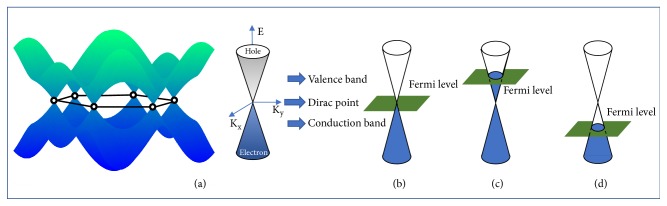
*Schematic diagram of energy band structure of graphene*. (a) Band structure of graphene. (b) Fermi level is at Dirac point. (c) Fermi level is in valence band. (d) Fermi level is in conduction band.

Thus, the thin layer conductivity of graphene is closely related to the Fermi level. In general, the concentration and type of carriers can be dynamically modulated by changing the Fermi level position of graphene.  Figure 4(a) is the band structure of grapheme [55, 56]. When the Fermi level is in the conduction band, the main carrier is free electrons; when the Fermi level is in the valence band, the main carrier is a hole; when the Fermi level is at Dirac point, the carrier concentration is at a minimum and the conductivity of graphene is also very low.

**Figure 5:**
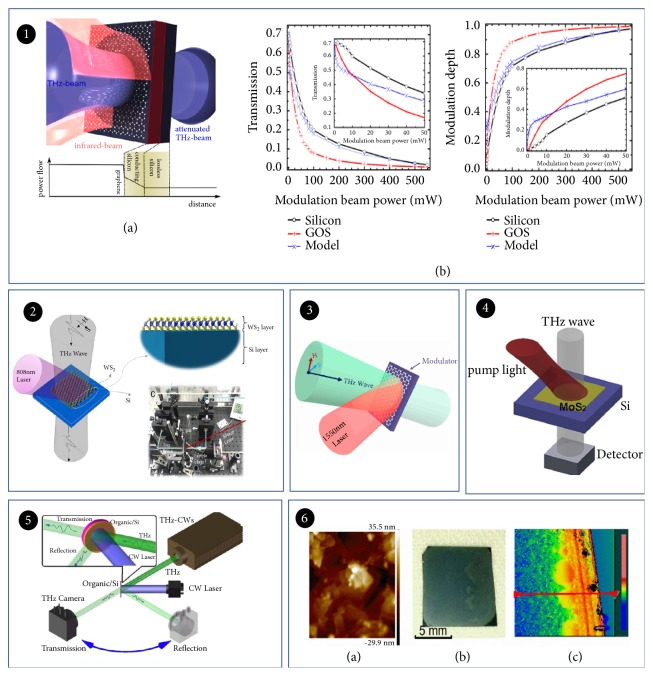
*Optically tuned THz modulator based on 2D materials*. Panel (1). (a) Schematic view of graphene on Si sample. (b) Normalized transmission (left) and depth (right) from the three phthalocyanine structures. Reproduced from [58]. Panel (2). Layer structure of the THz modulator based on WS_2_ and Si. Reproduced from [61]. Panel (3). The modulator consists of a single-layer graphene sheet on a germanium substrate. The beam of the THz wave is completely overlapped by the laser beam. Reproduced from [59]. Panel (4). A sketch map of the experiment. Reproduced from [62]. Panel (5). Experimental setup of the THz-CW for measuring transmission and reflection. Reproduced from [45]. Panel (6). (a) The AFM image of liquid-exfoliated WS_2_ nanosheets. (b) The image of the prepared WS_2_-Si sample. (c) Thickness distribution map of WS_2_ film measured by white light interferometer. Reproduced from [63].

Graphene has low insertion losses which is extremely ideal for optical modulation. Zhang Xiang's team firstly demonstrated it. They proposed an optical modulator by regulating the Fermi level of graphene [57]. The most graphene modulators consist of graphene and semiconductor material to form heterojunction. Laser beam penetrates Si to produce a large number of carriers; meanwhile, photogenerated carriers diffuse into the graphene layer and change its conductivity, which results in an amount of THz wave absorption by graphene layer. The most classic work is light-modulating graphene devices formed with graphene on Si (GOS) demonstrated by Weis in 2012 (Figure 5(1)). This modulator was pumped by a 780 nm femtosecond laser. The graphene layer absorbed the modulated beam of approximately 2.3%. As excited with wavelength of 750 nm and power of 40 mW pumped laser, this modulator could realize the modulation about 68%. The tunable THz bandwidth was in the range of 0.2-2 THz. When the optical pump energy reached 500 mW (@750 nm), the transmission THz wave almost completely disappeared [58]. By replacing Si with Ge, an optical modulator of full modulation based on graphene was demonstrated (Figure 5(3)). A laser with a wavelength of 1550 nm could be used to pump effectively. The measurement results presented that the modulation frequency range, modulation depth, and modulation speed were 0.25-1 THz, 94%, and 200 kHz, respectively [59]. Although it achieved full modulation, it required a high-power laser. With development of 2D material fabrication, an efficient THz modulator with low power was demonstrated. It needs only an external 450 nm continuous wave laser. It is mainly coated with a high-quality monolayer graphene film on a Si substrate. With external light excitation, the proposed modulator could reach a modulation depth of 74%. Incidentally, its modulation depth can be further improved [60].

Monolayer transition metal dichalcogenides (TMDs, such as MoS_2_ and WS_2_) are direct-bandgap semiconductors offering properties complementary to graphene [89]. A great number of modulators based on monolayer TMDs have been reported. For example, a bidimensional material modulator (Figure 5(4)) based on MoS_2_ and Si improved modulation depth as high as 64.9% at 0.9 THz pumped with an 808 nm laser. The modulation can be much higher after annealing MoS_2_. Under a larger pump power of 4.56 W, this depth was about 96%. These results suggested that MoS_2_ is a promising material [62]. Later on, many studies have been reported for improvement in terms of cost, manufacturing process, pump power, response speed, and modulation depth. Liu et al. studied a highly efficient active THz wave modulator based on MoS_2_/Ge structure. Monolayers of MoS_2_ and graphene samples were grown on* n*-doped Ge substrates by chemical vapor deposition (CVD). The light control bandwidth was greatly widened to reach 2.6 THz [90]. In addition to the expansion of the modulation bandwidth, Fan et al. demonstrated a THz modulator (Figure 5(2)) based on* p*-type annealed tungsten disulfide (WS_2_) and high-resistivity Si (*n*-type) structure. This modulator presented a laser power-dependent modulation mechanism. Ranging from 0.25 to 2 THz, the modulation depth reached 99% when the pumping laser was 2.59 W/cm^2^ [61]. Other researchers have adopted new methods to realize similar modulators. The size and thickness of WS_2_ film are controlled by innovatively depositing liquid-released WS_2_ nanosheets on Si instead of CVD method (Figure 5(6)). With raising the pump power, the modulation depth continues to increase, eventually reaching 94.8% under 470mW [63].

#### 2.1.3. Modulator Based on Flexible Substrate

**Figure 6:**
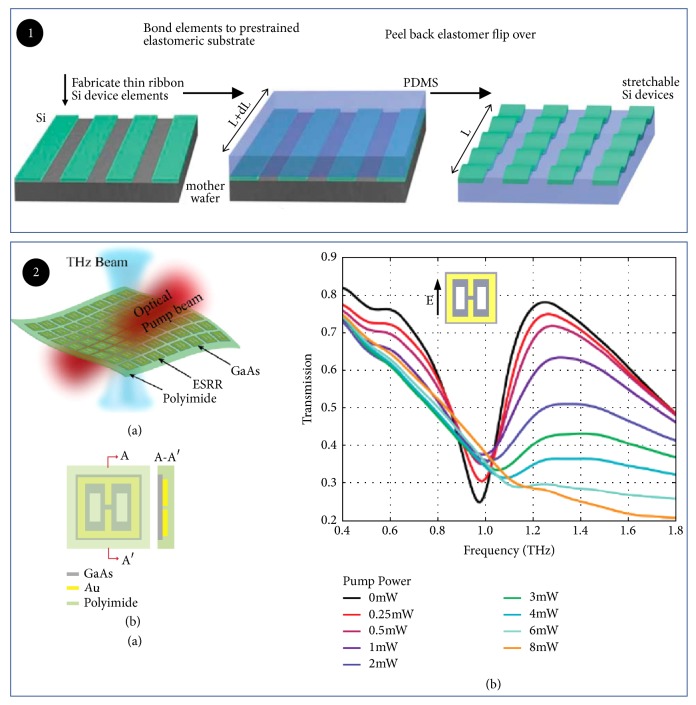
*Optically tuned THz modulator based on flexible substrate*. Panel (1). Schematic illustration of the process for building stretchable single-crystal Si devices on elastomeric substrates. Reproduced from [64]. Panel (2). (a) Schematic of the modulator structure. (b) Refractive index of the ferrofluid at different magnetic field intensities. Reproduced from [65].

Compared to rigid substrate THz modulators [91], flexible substrates have many advantages such as transparency, lightweight, low cost, and consistent adhesion [9, 92], which offer a new potential for THz modulator. For example, in 2006, D. Y. Khan et al. proposed a THz modulation on flexible substrates (Figure 6(1)). They made single-crystal Si with a thickness of 20 nm to 320 nm into a Si ribbon with a width of 5 *μ*m to 50 *μ*m and a length of 15 mm. When supported by a flexible substrate, this wavy Si ribbon could be reversibly stretched or compressed under high horizontal stress without damaging it. Considering the electrical properties of Si ribbon, its electrical parameters can be modified by changing the surface shape of the flexible substrate [64]. An optically tuned metamaterial modulator on a flexible polymer sheet, which had a frequency modulated in the THz range, was proposed by Liu et al. Electric split-ring resonators (eSRRs) are attached to a thin polyimide layer and assembled into the modulator (Figure 6(2)). The optical excitation of the GaAs patch changed the response of the metamaterial. The metamaterial effectively adjusted the effective dielectric constant. In their experiment, a modulation depth of 60% was achieved in the frequency range of 1.1-1.8 THz. This kind of flexible device can be more widely used and created on nonplanar structures for extensive applications in the future [65]. For current studies, high repeatability has not been obtained after multiple bending of the flexible substrate due to metal fatigue property [78, 93].

#### 2.1.4. Summary

The modulation properties based on different materials are summarized in Table 2. The material dominates the modulation depth and speed of THz wave, rather than the light source. Graphene and other 2D materials can achieve close to 100% modulation depth with modulation speed reaching the order of MHz. On the other hand, more experimental works should be carried out to explore those modulators based on simulations. The modulation efficiency of optically tuned THz modulator based on flexible substrate has yet to be improved. Lower insertion loss and more compact integration will be the future direction of optically tuned THz modulator.

### 2.2. Electrically Tuned THz Modulator

The other route for modulating THz wave is electrically tuned THz modulator. Using a mixed type-I/type-II GaAs/AlAs multiple-quantum-well sample, Libon et al. have demonstrated firstly an electrically controllable modulator in 2000 [94]. Electrical modulation is to control the concentration of electrons in the substrate materials or structures by applying bias voltage, which modulate the amplitude of the incident THz wave [94]. The transmission bandwidth and modulation depth and speed are limited by dielectric constant, loss, and response time of the materials. Consequently, to achieve high-speed band-pass modulation or broadband filter, it is necessary to find high-speed, low-loss THz functional materials and design novel structures.

**Figure 7:**
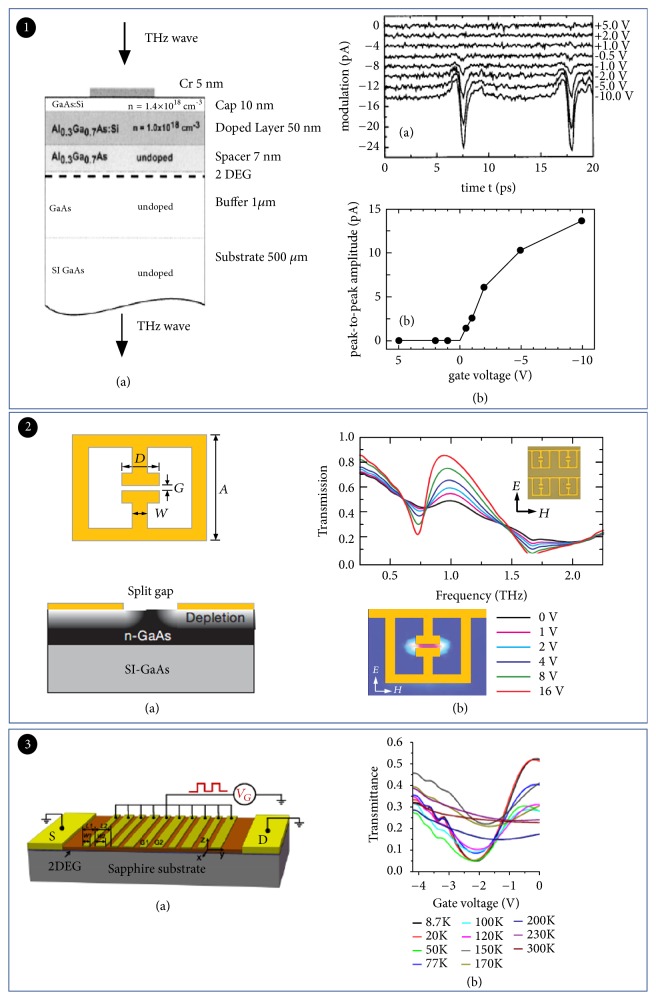
*Electrically tuned THz modulator based on semiconductors and metamaterials*. Panel (1). (a) The structure of the THz modulator. (b) Gate voltage dependence of the modulation signal (upper). Peak-to-peak amplitude of the modulator signal vs. gate voltage (nether). Reproduced from [66]. Panel (2). (a) Geometry and dimensions of the THz metamaterial switch/modulator. (b) Frequency-dependent transmitted intensity of THz radiation. Reproduced from [8]. Panel (3). (a) Three-dimensional schematic illustration of the plasmon-based THz modulator. (b) Dependence of transmission on gate voltage at different temperatures. Reproduced from [67].

Similar to the main idea of optically tuned THz modulator, charge injection can also modulate the THz wave. In the past decade, 2D electron gas (2DEG) in HEMTs has been demonstrated to modulate THz wave effectively. HEMT is a field effect transistor that provides a 2DEG quantum well at the heterojunction interface of a highly doped semiconductor (typically AlGaAs) and an original undoped semiconductor (GaAs). In 2000, Kersting presented a GaAs/AlGaAs heterostructure device which powered up the phase modulation of THz signals [94]. This modulator contained five parabolic quantum wells (PQWs). THz wave modulation can be realized by stimulating electrically low-energy electron to high-energy states.  Figure 7(1)(b) shows the power spectrum of the differential modulation signal and the power spectrum of the incident pulse. It led to the development of THz electronics chip due to a reduction of the device dimensions. Nevertheless, it cannot operate at low temperatures. To allow THz modulator to be operated at room temperature, Kleine-Ostmann et al. presented room-temperature THz wave modulator by using a device based on a gated 2DEG [66]. This modulator mainly included a 2DEG whose density could be controlled by the gate voltage. This major breakthrough illustrated that 2DEG in semiconductors could be used to control THz wave effectively [95]. Similarly, it has also been used for THz emission [96], THz detection [97], etc. Other 2DEG systems can be also used to realize the modulation; for example, a THz modulator based on 2DEG of a GaN/AlGaN heterostructure was demonstrated by Zhou (Figure 7(3)). The modulator had a maximum intensity modulation depth of 93% and a 3dB operating bandwidth of 400 kHz. It required only a low driving voltage amplitude of 2V under 8.7 K. This active plasma-based THz modulator may provide a promising solution in THz technology fields for the metamaterial THz modulator [67].

#### 2.2.1. Modulator Based on Semiconductors and Metamaterials

In addition, metamaterials are also a hot spot in THz modulator [98]. Kebin Fan et al. demonstrated a metamaterial active device which consists of gold split-ring resonators (SRRs) on GaAs thin film grown on Si substrate [8]. It has achieved 50% modulation depth and 100 kHz modulation speed. Schottky/n-doped GaAs devices had been developed to increase greatly the modulation speed. In further studies, the modulation speed had reached 10 MHz; however, the modulation depth of this modulator was only 30% [99]. The single-particle nonresonant absorption mechanism described in Drude model has been used in the related studies. As seen from experiments mentioned above, Drude model has not yet been effective enough for the theory of metamaterial modulator [100].

#### 2.2.2. Modulator Based on Graphene

To further improve the modulation depth and modulation speed of THz modulator, different active medium as substrate has been tested. The refractive index of those materials can usually be written in the form of a complex refractive index: *n* = *n*_*r*_ + *in*_*i*_, where *n*_*r*_ is the real part of the refractive index and *n*_*i*_ the imaginary part of the refractive index. When the voltage is adjusted, the real and imaginary parts are usually changed at the same time. By using those changes, different electric modulators can be developed. For example, electroabsorption modulator is based on imaginary part changing. When the real part of the refractive index changed with applied voltage, the transmission phase of THz wave changed. Therefore, for the electroabsorption modulator based on 2D material, a better modulation effect can be obtained by changing the refractive index through applied voltage. In fact, electromodulators based on 2D materials mainly focused on graphene. Graphene has many particular properties, such as adjustable thin layer conductivity and long mean free path, due to its unique conical energy band structure [101–103]. Its linear dispersion relationship between energy and crystal momentum makes graphene superior to other semiconductor materials.

**Figure 8:**
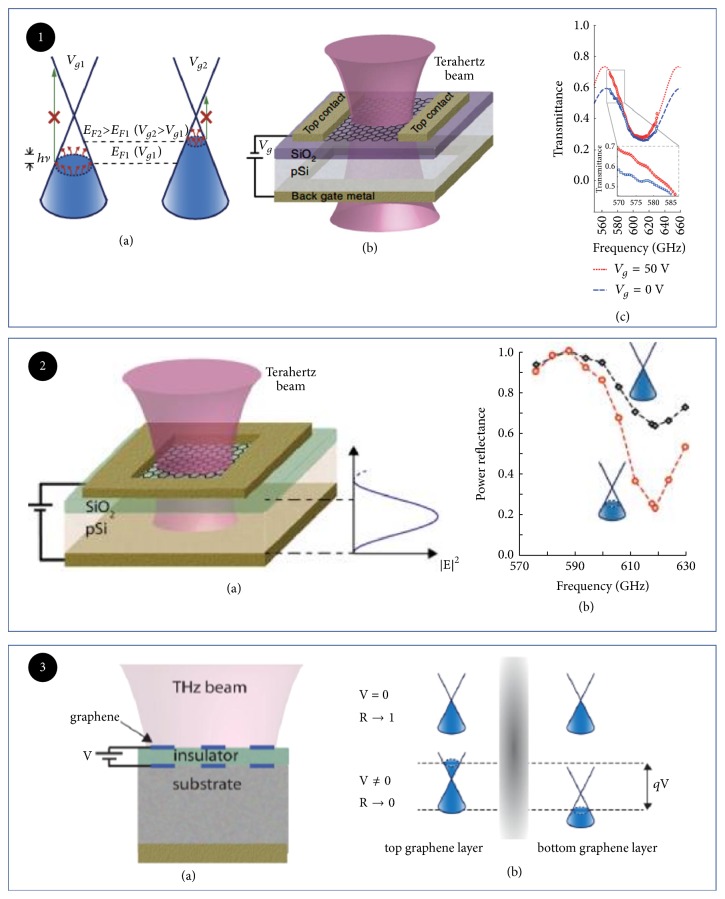
*Electrically tuned THz modulator based on graphene*. Panel (1). (a) Schematic of the proof-of-concept graphene THz modulator. (b) Schematic of the THz modulator. (c) Schematic of the device, and electric field distribution when the substrate thickness and plasmonic resonance are matched to an odd multiple of a quarter wavelength of the THz wave. Reproduced from [68]. Panel (2). (a) Schematic of the THz modulator. (b) Normalized reflectance (R/R (VCNP)). Reproduced from [69]. Panel (3). (a) Schematic of the device, and electric field distribution. (b) Operation mechanism of the self-gated graphene pair. Reproduced from [70].

S. Rodriguez utilized graphene to modulate THz wave (Figure 8(1)). The modulation depth and modulation speed could reach 15% and 20 kHz, respectively [68]. Meanwhile, the same research group presented a reflective THz modulator, as shown in Figure 8(2). The silver film on the back of the reflective THz modulator acted as both gate and mirror, which improved the modulation depth with 64% and expanded bandwidth ranging 0.57-0.63 THz compared to their early works. However, its insertion loss and modulation speed only reach 2 dB and 4 kHz, respectively [69]. The essence of those works is to change the Fermi level of graphene by adjusting applied voltage. The following graphene THz modulators with different structures are also based on this method. To improve modulation depth, multiple layers of graphene are a choice [102]. As shown in Figure 8(3), a THz electroabsorption modulator based on two-layer graphene with periodic micron-belt pattern was presented. The THz wave was vertically illustrated on the surface of graphene, which enhanced the absorption of light because of the plasma effect. This modulator could operate at frequencies up to tens of THz, and the modulation depth could even reach 100% [103].

**Figure 9:**
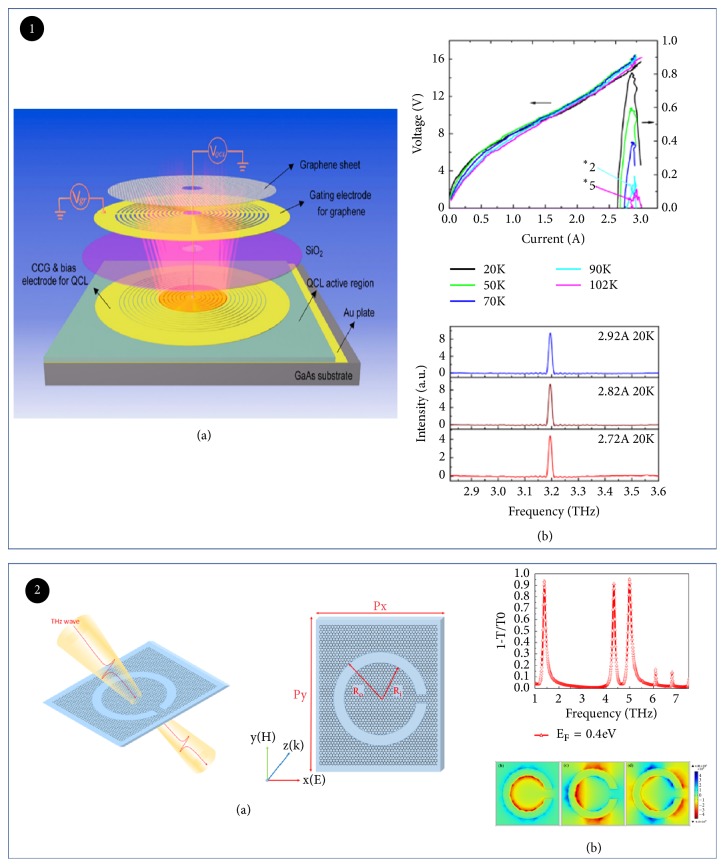
*Electrically tuned THz modulator based on graphene*. Panel (1). (a) Overview of the quantum cascade laser integrated graphene modulator. (b) Light-current-voltage (LIV) characteristics of the THz CCG QCL at different heat sink temperatures. (Upper) Laser spectra as a function of pump current I. (Nether) Reproduced from [71]. Panel (2). (a) Right: schematic of the light interacting with graphene CSRR. Left: schematic of the graphene CSRR. (b) Extinction spectrum in transmission of the graphene CSRRs (upper). Distributions of normalized z-component of electric field* Ez* at 1.38THz, 4.33THz, and 5THz, respectively (nether). Reproduced from [72].

Usually, the modulation depth of most modulators based on monolayer graphene can only reach 80% [69]. However, monolayer graphene combined with other structures or devices can improve the modulation depth. For example, G. Liang puts monolayer graphene on top of quantum cascade laser (QCL) to demonstrate modulation depth of 100% (Figure 9(1)) and modulation speed of 110 MHz [71]. The high modulation depth was a consequence of a strong interaction between the graphene and THz wave, whereas the high modulation speed can be improved by reducing the dimension of device. It has recently been demonstrated that a THz modulator can realize full modulation with a wide modulation bandwidth and fast modulation speed. Using monolayer graphene, Huang proposed an adjustable complementary ring resonator (Figure 9(2)). It was worth mentioning that in the range of 1-2 THz and 3-7 THz, three significant resonant peaks can be modulated. The maximum modulation depth reaches 98.8% at 7.47 THz [72].

#### 2.2.3. Modulator Based on Flexible Substrate

**Figure 10:**
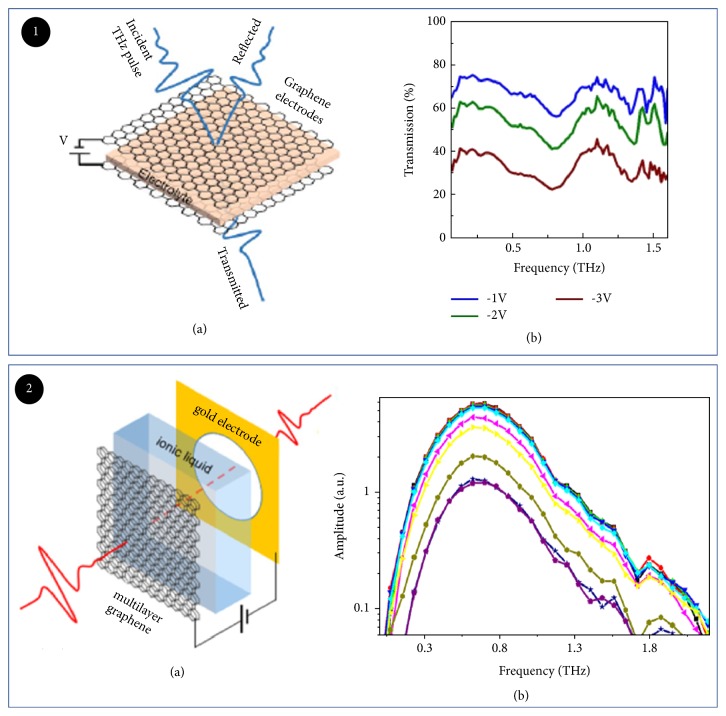
*Electrically tuned THz modulator based on flexible substrate*. Panel (1). (a) Schematic representation of the graphene supercapacitor used as a broadband THz modulator. (b) Spectrum of the transmitted THz signal obtained after Fourier transformation of the recorded signal and normalization. Reproduced from [73]. Panel (2). (a) THz setup and MLG structure (inset) consisting of MLG sandwiched between host (PVC or PE), electrolyte, and gold electrode. (b) Corresponding frequency domain amplitudes. Reproduced from [74].

Flexible substrate was used to fabricate optical modulator with many merits [104, 105] as described in Section 2.1.3. For electric modulator, flexible substrate plays also an important role. For example, Kocabas et al. presented a graphene metamaterial THz modulator based on a flexible substrate (Figure 10(1)), which consists of two large-area graphene electrodes transferred onto THz transparent substrates with ionic liquid electrolytes between them. The bias can change electrostatic doping of graphene, which affects its optical properties. In this case, the maximum modulation depth reaches only 50% at 0.1-1.4 THz working at a bias of 3V. The modulation speed was slow [73]. Virtually, this modulator showed excellent flexibility, which has not been deformed after being flexed many times. Modulator based on flexible substrate can also realize a high modulation depth. For example, E. Kaya et al. have demonstrated that polyvinyl chloride (PVC) and polyethylene (PE) were selected as flexible substrates (Figure 10(2)). A multilayer graphene modulator was fabricated on those flexible substrates by chemical vapor deposition. This device could fully modulate THz wave with a frequency range of 0.2-1.5 THz at a low voltage of 3.4V [74]. The comparison of electrically tuned modulation properties based on different substrate materials is shown in Table 3.

#### 2.2.4. Summary

The modulation properties of different materials reported in literatures are given in Table 2. Graphene can achieve modulation depth close to 100% and the modulation speed in the order of kHz. At present, there are few reports on flexible THz modulators since organic functional materials and flexible substrates cannot fully be compatible with micronanofabrication processes. Based on reviewing the electric modulator, it implied that electric modulation device needs to miniaturize the structure, to low insertion loss, and to improve modulation speed, which will be the future direction.

### 2.3. Photoelectric Hybrid Tuned THz Modulator

Sections 2.1 and 2.2 described the modulation methods using electricity or light separately. If two methods can be combined together, better modulation effects may be obtained. The photoelectric hybrid modulation method is a method of synthesizing electric and optical modulation. Its basic principle is using pump light excited carriers in a base semiconductor material. In the meantime, the motion direction of the carrier is adjusted by an external bias [108]. For example, an optoelectric hybrid modulator was exhibited by Q. Li et al. [75]. Its structure was shown in Figure 12. Si substrate was pumped with a continuous laser of 532 nm to generate a large number of electron-hole pairs. Under the drive of the concentration difference, the photoelectrons in the Si diffused into the graphene until the equilibrium state was established. A structure similar to a PN junction was formed between the two materials. When the bias was varied from 0 V to -3 V, the peak modulation of the time-domain signal peak achieved 51% at optical pump power with 420 mW. When the bias changed from 0 V to -4 V, the modulation amplitude depth became 83%. Based on this structure, Ran Jiang et al. used a Si:HfO_2_ material added between graphene and Si substrate to realize a device that had a modulation effect under both biasing directions [76]. However, it is more difficult to implement a hybrid modulation method since there are completely different responses when applying positive or negative biases. Quan Li et al. realized an active modulation at low voltage (~1V) by a photoelectric hybrid modulator with a two-dimensional material on a structured Si substrate [77].

As a new modulation method, photoelectric hybrid tuned THz modulator has not drawn much attention. However, this extreme high sensitive method is in need urgently.

### 2.4. Mechanically Tuned THz Modulator

**Figure 11:**
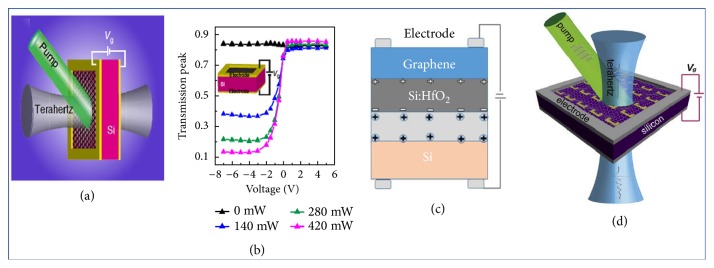
*Photoelectric hybrid tuned THz modulator*. (a) Illustration of the GSTD sample. The double-layer graphene on the Si substrate was photoexcited with green light and biased with voltage V_g_. (b) Gate voltage-dependent, normalized time-domain transmission peaks of the double-layer graphene on Si. (c). Schematic drawings of working mechanism for the GSS structure. (d) Monolayer graphene deposited on the SRRs with continuous wave laser light excitation and bias voltage Vg. Reproduced from [75–77].

**Figure 12:**
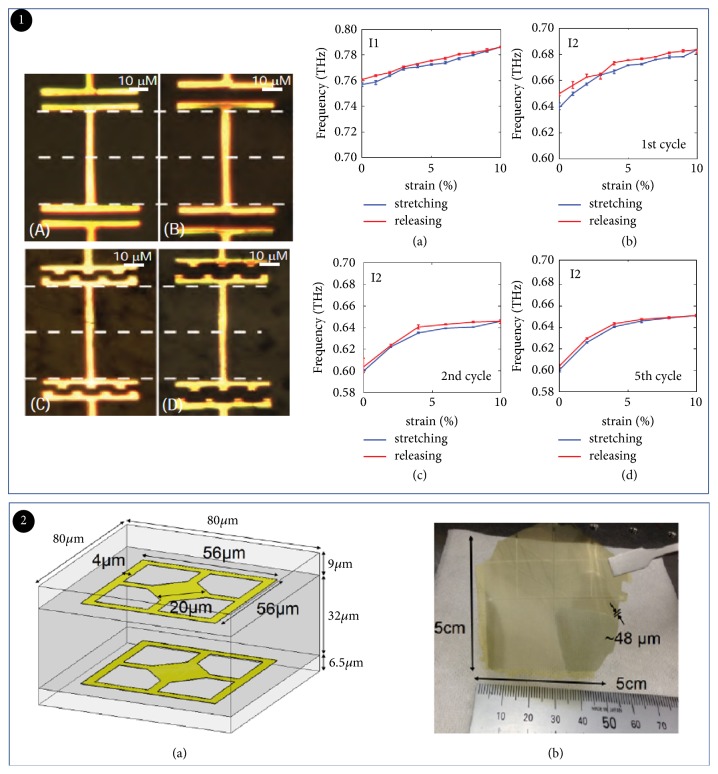
*Self-deformation tuned THz modulator*. Panel (1). THz modulator structure diagram (a) and modulation performance diagram (b) based on metamaterial. Reproduced from [78]. Panel (2). (a) Schematic of the film metamaterial unit structure with double-layer USRRs for THz wave phase shifter. (b) Overview of the developed flexible film metamaterial device. Reproduced from [79].

THz modulators on flexible substrates have been introduced in Sections 2.1.3 and 2.2.3. These devices can also be applied to complex nonplanar surfaces such as communications fibers, aircraft, and radar surfaces [75, 109]. Alternatively, the modulation of THz wave was achieved by preparing metal metastructures on the surface and changing space between them [110]. For example, in 2013, Li et al. presented a flexible and tunable THz metamaterial (Figure 11(1)). The tensile deformation of substrate caused change of the pitch, which in turn affected the transmission of THz waves. Thus, this THz device can be used to detect the deformation of different objects. When the deformation appeared multiple times, there were metal fatigue phenomena which affected the stability. This method can also achieve phase modulation. For example, an ultrathin THz wave phase shifter was described by Z.L. Han [79]. Each metamaterial unit consists of double-layered structure. The distance change between two layers affects phase shift which caused phase delay. This phase shifter has high transmission with a coefficient of 91%. Compared to traditional THz wave phase shifters, this ultrathin flexible phase shifter has better signal transmission or reflection (Figure 11(2)). It could be integrated with other systems to improve device adjustability. These findings paved the way for flexible THz electronics and contributed to the development of THz technology [111].

Nowadays, flexible electronic devices have gradually become a research hotspot with the development of wearable electronic devices, flexible displays, and health monitoring devices. One type of modulator affects the intensity variation of the THz wave by its self-deforming [112]. Unfortunately, there are no reports on THz modulation devices with high stability in the deformed state [113]. Therefore, studying a THz modulator with stable modulation performance under flexible deformation is a problem to be solved.

### 2.5. Thermally Tuned THz Modulator

**Figure 13:**
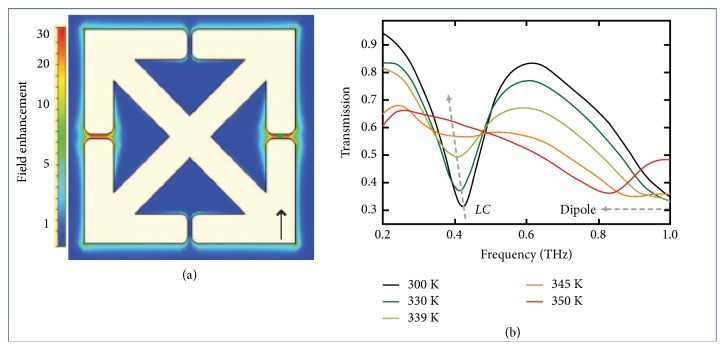
*Thermally tuned THz modulator*. (a) Resonant field enhancement as a function of position. (b) Temperature-dependent THz transmission spectra of SRRs on VO_2_. Reproduced from [80].

In many transition metal oxides, the modulation of THz wave is achieved by external stimuli, including temperature, light, electric field, mechanical strain, or magnetic field [80, 114, 115]. Temperature changes can affect the mobility and lifetime of free carriers in those materials. Vanadium dioxide (VO_2_) film is a metal oxide with an insulator-metal phase transition property, which can be converted from insulating state (monoclinic structure) to metallic state (tetragonal structure) under heat [116, 117]. It leads to reversible mutation in physical properties [118, 119]. Both theory and experiment have exhibited that VO_2_ film has high transmittance in the insulating phase and opposite property in the metal phase [120]. Therefore, VO_2_ is a good thin film phase change material suitable for the THz modulator (Figure 13) which consists of periodic metastructure on the surface of VO_2_; then THz wave can be modulated by controlling the temperature [121].

### 2.6. Magnetically Tuned THz Modulator

**Figure 14:**
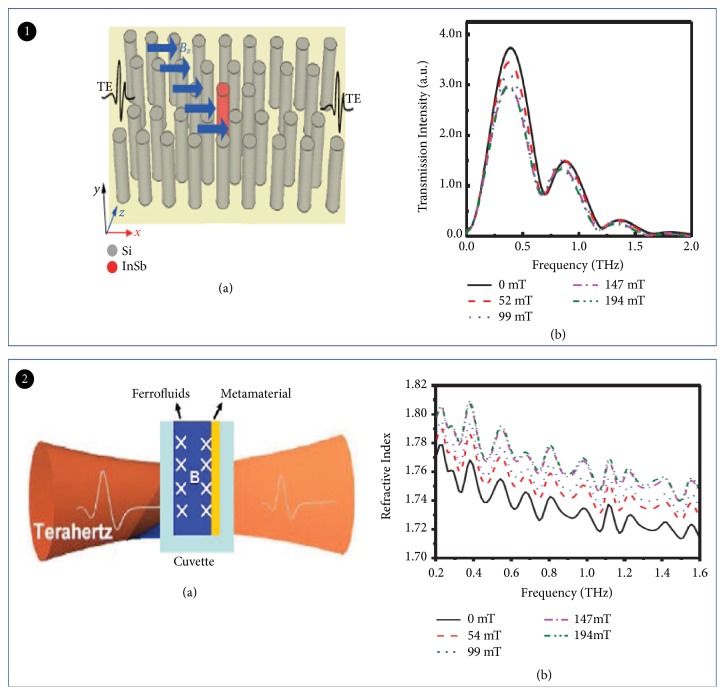
*Magnetically tuned THz modulator*. Panel (1). (a) The structure model of THz wave modulator based on magnetized plasma PC. (b) The real part (solid) and image part (dotted) of neffof InSb in the THz regime with the dependence of the external magnetic field. Reproduced from [81]. Panel (2). (a) Schematic of the modulator structure. (b) Refractive index of the ferrofluid at different magnetic field intensities. Reproduced from [82].

Magnetic-tuned terahertz modulator was based on the magnetooptical effect under an external magnetic field. For example, magnetized plasma 2D photonic crystal THz wave modulator was presented by Wen (Figure 14(1)) [81]. The resonant frequency can be tuned with the insertion loss of 0.3 dB. The modulation speed was as high as 4 GHz. Thus, this modulator has the potential for THz wireless broadband communication system.

A THz wave modulator based on Fe_3_O_4_-nanoparticles was reported recently (Figure 14(2)) [82]. This modulator consists of a magnetic fluid and metamaterial structure. The modulator confirmed a 34% modulation depth. It is worth mentioning that metamaterials cause a 33 GHz frequency shift at low magnetic field. This modulator will have many potential applications in THz filtering, modulation, and sensing.

Although magnetically tuned THz has more applications, unfortunately, they are poorly controllable in the current study.

### 2.7. MEMS Tuned THz Modulator

**Figure 15:**
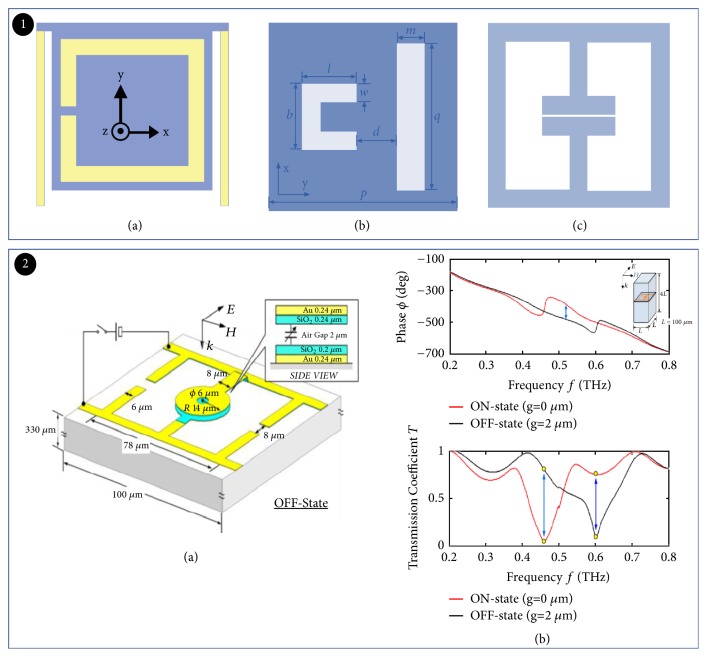
*MEMS tuned THz modulator*. Panel (1). Schematic illustration of unit cell. Reproduced from [83–85]. Panel (2). (a) MEMS reconfigurable SRR design. (b) Tunable THz switch performance phase (upper) and transmission amplitude (nether). Reproduced from [86].

Microelectromechanical systems (MEMS) are a high-tech frontier discipline in modern information technology [122–124]. The continuous improvement of MEMS technology provides superiority for THz modulator. For example, H. Tao et al. [83] achieved modulation of the resonant intensity in the THz band (0.5 THz) based on thermal drive technology in 2009. Q. Bai [84] et al. demonstrated a planar semiconductor metamaterial device utilizing MEMS-based slow-light tunable effects, which could tune over a wide frequency range in the THz band. Ozbey and Aktas [85] presented THz metamaterial device that used magnetically driven resonant frequency (Figure 15(1)). Z. Han demonstrated a reconfigurable metamaterial structure fabricated by MEMS technique [125]. The resonant frequency can be tuned by using the voltage to control the height of the center metal ring (Figure 15(2)). The modulation range was relatively narrow, which could only achieve modulation between 0.45 THz and 0.65 THz [86]. According to the merit of MEMS technology, the MEMS tuned THz modulator with high modulation efficiency could achieve extensive applications in high-power field.

### 2.8. THz Modulator Based on Coding and Programmable Metamaterials

**Figure 16:**
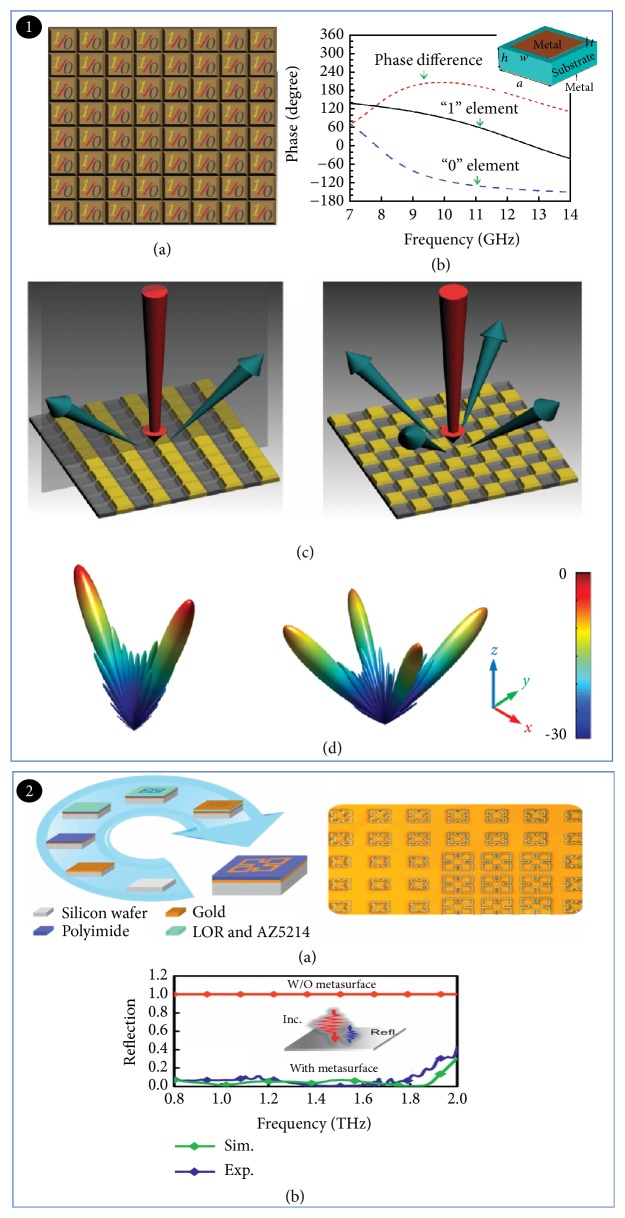
*THz modulator based on coding and programmable metamaterials*. Panel (1). The 1-bit digital metasurfaces and coding metasurfaces. (a) Two types of elements: “0” and “1.” (b) Different phase responses of two elements in a range of frequencies. (c) Different encodings produce different modulation effects. (d) Simulation calculation result. Reproduced from [87]. Panel (2). The fabrication and measurement results of the coding metasurfaces. (a) The fabrication process for the coding metasurfaces. (b) The measured and simulated backward scattering coefficients of the 2-bit coding metasurfaces. Reproduced from [88].

The unique electromagnetic properties of electromagnetic metamaterials result in rapid evolving. For example, in 2014, Giovampaola and Engheta proposed a method of constructing metamaterials through spatially mixed “digital metamaterial bits” [126]. This new concept metamaterial can be used in THz modulator. At the same year, Tie Jun Cui et al. proposed the concept of coded metamaterials [87]. They abandoned the traditional method of effective medium theory and designed the encoded metamaterials of 1-bit code sequences, which were used to flexibly modulate THz waves. As shown from Figure 16, the “0” and “1” elements represent the ideal magnetic and electrical conductors, respectively. The phase difference after reflection is 180°. According to the traditional phase-array-antenna theory, the scattering pattern on the metasurfaces under the coding sequence can be calculated to design different coding sequences. Take the coded metasurfaces composed of N×N square grids with dimension D as an example [87, 127].

(6) $$\begin{array}{c} dir\left(\theta ,\varphi \right)=\frac{4\pi {\left|f\left(\theta ,\varphi \right)\right|}^{2}}{\int_{0}^{2\pi }\int_{0}^{\pi /2}{\left|f\left(\theta ,\varphi \right)\right|}^{2}\sin\theta d\theta d\varphi } \end{array}$$

where *θ* and *φ* are the elevation and azimuth angles, respectively, and *f*(*θ*, *φ*) is the pattern function of a lattice. That is to say, different encodings produce different modulation effects. When the normal incident wave illuminates the metasurfaces, two and four reflected beams are formed (Figure 16), which illustrate that metasurfaces can modulate the THz wave. A 2-bit random coding arrangement was used to obtain the expected low-scattering pattern, which achieved wide-band diffuse reflection of terahertz waves [88]. Shuo Liu et al. proposed the concept of anisotropically encoded metamaterials in 2016 [128]. Their function depends on the polarization direction of the incident wave. The beam splitter used in metasurface can separate the orthogonal polarization modulated terahertz signals, which can be used to increase the transmission rate in ultra-high-speed wireless communication in the future. Coding and programmable metamaterials, a new approach to study and design from the information perspective, provide us with great flexibility in controlling the radiation of the electromagnetic wave in both amplitude and phase [129]. The research results illustrate that programmable metamaterials are a possible route to implement more functional THz modulators in the future.

## 3. Conclusions and Outlook

We have extensively reviewed modulators operating in THz frequency range and summarized the principles, current status, and advantages and disadvantages of various methods.

At present, electrical and optical modulation THz devices have achieved exciting results in terms of modulation depth and modulation speed. Compared with electrical modulation, optical modulation has deeper modulation depth, faster modulation speed, and easier modulation method. Although the photoelectric hybrid modulation can be more flexible, the modulation method is obviously complicated. Other methods provide new attempts for THz modulation; however, the extension and simple implementation in the frequency range need further study. In terms of material selection, 2D materials and flexible substrates have become the focus of recent experimental research due to their huge advantages. Programmable metamaterials dynamically control the phase responses for each element. It can be considered as a possible route for the future realization of THz modulators with far more functionalities.

THz modulator research is a dynamic, fast-growing, and challenging field. An ideal modulation device needs a higher degree of miniaturization, lower insertion loss, and higher modulation speed, which will be the core of building a THz communication system. More explorations are required for various actual devices to get an excellent modulation effect.

## Figures and Tables

**Table 1:** The comparison of different carrier communication characteristics.

Carrier	Microwave	THz	Optical
	communication	communication	communication
Transmission mode	Wireless networks	Wireless networks	Wired networks
Transmission distance	Long range	Visual range	Ultra-long range
Message capacity	Mbit/s	Between	Gbit/s
Directionality	Bad	Between	Fine
Security	Low	Low-radiation	High photon energy

**Table 2:** The comparison of optically tuned modulation properties based on different materials.

Work	Description	Year	Frequency	Modulation depth	Ref.
Libon et al.	Multiple quantum well	1999	0.2-1 THz	40%	[34]
Deng L. et al.	InSb gratings	2013	1.5 THz	46.70%	[42]
Peter W. et al.	Graphene on Si (GOS)	2012	0.2-2 THz	99%	[56]
Wen Q. Y. et al	Graphene on Ge	2014	0.25-1 THz	94%	[57]
Cao Y. et al.	MoS_2_	2016	0.5- 1.5THz	96%	[62]
Fan Z.Y. et al.	WS_2_	2017	0.25- 2 THz	99%	[61]
Hyung K. Y.et al.	C_60_	2014	0.5- 1.5THz	98%	[43]
He T. et al.	AlClPc	2015	0-2.5 THz	99%	[45]
Shi Z. et al.	Si Nanotip	2017	0.25-1 THz	>90%	[44]

**Table 3:** The comparison of electrically tuned modulation properties based on different substrate materials.

Work	Description	Year	Frequency	Modulation depth	Ref.
Huang Y. D.	Plasmonic	2016	-	93%	[67]
Zhou G.	VO_2_	2017	0.3-1.0THz	>50%	[100]
Sensale-R. B.	Graphene	2012	10THz	100%	[106]
Liang G.	Graphene	2015	3THz	94%-100%	[71]
Huang Z.	Monolayer Graphene	2018	1-7THz	>85%	[72]
Chikhi N.	Liquid crystals	2018	1-3THz	>90%	[107]
Kaya E.	Flexible Substrate	2018	0.2-1.5THz	100%	[74]
